# Supplementary material for: Psychological distress among parents with emigrant offspring: A mixed-methods study from Changunarayan Municipality, Nepal
Source: PLoS One. 2025 Aug 6;20(8):e0329071. doi: 10.1371/journal.pone.0329071 (PMC12327691; doi:10.1371/journal.pone.0329071)
Supplement: S1 Table — (DOCX) [file pone.0329071.s001.docx]

**Supporting information:**

**S1 Table: Descriptive Statistics of Kessler Psychological Distress Scale (K10) Scores**

|  | | Statistic | Std. Error |
| --- | --- | --- | --- |
| Mean | | 15.25 | .449 |
| 95% Confidence Interval for Mean | Lower Bound | 14.36 |  |
|  | Upper Bound | 16.13 |  |
| 5% Trimmed Mean | | 14.47 |  |
| Median | | 13.00 |  |
| Variance | | 43.984 |  |
| Std. Deviation | | 6.632 |  |
| Minimum | | 10 |  |
| Maximum | | 41 |  |
| Range | | 31 |  |
| Interquartile Range | | 8 |  |
| Skewness | | 1.633 | .165 |
| Kurtosis | | 2.320 | .328 |
