# Supplementary material for: Psychological distress among parents with emigrant offspring: A mixed-methods study from Changunarayan Municipality, Nepal
Source: PLoS One. 2025 Aug 6;20(8):e0329071. doi: 10.1371/journal.pone.0329071 (PMC12327691; doi:10.1371/journal.pone.0329071)
Supplement: S2 Table — (DOCX) [file pone.0329071.s002.docx]

**Supporting information:**

**S2 Table: Bivariate Analysis of Continuous K10 Scores Across Participant and Migration-Related Characteristics**

| **Independent factor** | **Categories** | **n** | ***Median (IQR)*** | ***p*-value** |
| --- | --- | --- | --- | --- |
| Sex | Male | 80 | 12 (5) | **0.038 #** |
|  | Female | 138 | 13.5 (8) |  |
| Ethnicity | Dalit | 3 | 27 (-) | 0.276 + |
|  | Disadvantaged janajati | 16 | 13 (6) |  |
|  | Relatively advantaged janajati | 36 | 12.5 (8) |  |
|  | Brahmin/ Chhetri | 163 | 13 (*) |  |
| Marital status | Married | 190 | 12 (8) | **0.009 #** |
|  | Widow/Widower | 28 | 17 (6.8) |  |
| Education Level | Illiterate | 23 | 16 (11) | 0.071 + |
|  | Literate through informal education | 57 | 13 (9) |  |
|  | Primary Level (1-8) | 43 | 13 (9) |  |
|  | Secondary Level (9-10) | 59 | 12 (4) |  |
|  | Higher Secondary Level (11-12) | 20 | 14 (8) |  |
|  | Bachelor and higher degrees | 16 | 11 (5) |  |
| Occupation | Homemaker | 76 | 13 (8) | 0.104 + |
|  | Agriculture | 53 | 14 (9) |  |
|  | Retired | 42 | 11 (4) |  |
|  | Business | 38 | 13 (8) |  |
|  | Service | 9 | 10 (6) |  |
| Chronic Conditions | None | 121 | **12 (6)** | **0.008 +** |
|  | Single morbidity | 49 | 14 (8) |  |
|  | Multi morbidities | 48 | 15 (13) |  |
| Support from Significant Other | Low Support | 38 | **16 (15)** | **0.001 #** |
|  | Moderate/High Support | 180 | 12 (8) |  |
| Support from Migrant Child | Low Support | 16 | 16 (15) | **0.033 #** |
|  | Moderate/High Support | 202 | 12 (8) |  |
| Support from Friends | Low Support | 96 | 14 (9) | 0.204 # |
|  | Moderate/High Support | 122 | 12 (7) |  |
| Number of Emigrant Children | Single child | 181 | 12 (8) | **0.035 #** |
|  | More than one child | 37 | 14 (11) |  |
| Age of Emigrants | <25 years | 61 | 12 (7) | 0.340 + |
|  | 25-30 years | 71 | 14 (8) |  |
|  | >30 years | 86 | 14 (8) |  |
| Sex of Emigrants | Male | 148 | 13 (8) | 0.901 # |
|  | Female | 70 | 12.50 (8) |  |
| Duration of Migration | Recent migration (1 year) | 47 | 14 (10) | 0.316 # |
|  | Long-term migration (≥2 years) | 171 | 12 (7) |  |
| Country of Migration | Australia | 69 | 12 (5) | 0.070 + |
|  | Golf countries | 66 | 13.5 (8) |  |
|  | USA/Canada | 49 | 13 (8) |  |
|  | UK/Europe | 34 | 15 (10) |  |
| Nature of Visa | Working visa/ dependent visa | 103 | 15 (9) | **0.012 +** |
|  | Student visa | 66 | 12 (6) |  |
|  | Permanent resident | 49 | 12 (5) |  |
| Type of Visa | Temporary | 169 | 12 (5) | 0.051 # |
|  | Permanent | 49 | 14 (8) |  |
| Occupation of an Emigrant | Jobs | 164 | 13 (8) | 0.105 + |
|  | Student | 47 | 12 (8) |  |
|  | Labor | 7 | 22 (21) |  |
| Financial Support from Emigrant | Yes | 152 | 12 (7) | 0.219 # |
|  | No | 66 | 13 (10) |  |
| Frequency of Communication with Emigrants | Daily | 108 | 12 (7) | 0.395 + |
|  | Weekly | 96 | 14 (9) |  |
|  | Monthly | 14 | 13.50 (8) |  |
| Frequency of Home Visit | Frequent (in every 1-2 years) | 147 | 14 (9) | 0.172 # |
|  | Infrequent | 71 | 12 (7) |  |

*#Mann-Whitney U test, +Kruskal-Wallis H test; Bold p-value signifies statistical significance at p<0.05*
