# Supplementary material for: Psychological distress among parents with emigrant offspring: A mixed-methods study from Changunarayan Municipality, Nepal
Source: PLoS One. 2025 Aug 6;20(8):e0329071. doi: 10.1371/journal.pone.0329071 (PMC12327691; doi:10.1371/journal.pone.0329071)
Supplement: Attachment — Submitted filename: PLOS ONE 05072025 comments to authors.docx [file pone.0329071.s006.docx]

PLOS ONE

Psychological Distress among Parents with Emigrant Offspring: A Mixed-Methods Study from Changunarayan Municipality, Nepal

**Summary and Overall Impression**

This study used mixed methods to explore psychological distress among older parents whose children had emigrated from Nepal. The study identified factors associated with greater distress, including multiple morbidities, low support from emigrant children, etc. Overall the study explored an important and somewhat understudied topic. It was well done and well written.

**Major Issues**

- Avoid use of “elderly” as it’s somewhat age biased language. Also, 2.8% were aged 30-40 years and 34.9% were 40-50 years
- It would be good to have more detail about the financial support from emigrant children. While not statistically significant (<.05) according to the chi-square test in Table 5, the p-value of .084 I think warrants exploring in the regression model. There may be possible interactions too that could be explored. It may be part of the reason that being employed as a laborer was associated with psychological distress.
- I wonder if the authors considered analyzing psychological distress as a continuous variable? There is some loss of data when recoding into categories.

**Minor Issues**

- Age categories in Table 3 are overlapping
- In first paragraph of the discussion, the authors state “The qualitative findings revealed a complex emotional landscape, where some parents reported distress and loneliness, while others expressed pride in their children's achievements.” They should acknowledge that it’s possible for the same parents to have ambivalent feelings.
- Several places where a space is needed before an in-text citation
- Authors should clarify what diseases/conditions were considered
- On page 9 in the paragraph starting with “Parental well-being is deeply influenced by the migration of their children, often resulting in emotional and psychological challenges” The authors cite several studies of the prevalence of distress among left-behind parents, but I wonder if the rates are higher compared to those who do not have emigrant children? It seems some of the studies look at this, but more detail would be helpful about studies cited in the second half of the paragraph. This should also be clarified in the first paragraph of the discussion section.
- The data have already been collected, but it would have been interesting to examine the potential relationship between emigrant children’s long-term plans (to return or not) and parents’ psychological distress. You note this in the limitations section but I wonder if you noticed any patterns in the qualitative data?
- One of the review questions is “Have the authors made all data underlying the findings in their manuscript fully available?” If the authors have, they should include a statement about this in the methods section.
